# Effects and Mechanism of Oxymatrine Combined with Compound Yinchen Granules on the Apoptosis of Hepatocytes through the Akt/FoxO3a/Bim Pathway

**DOI:** 10.1155/2022/8644356

**Published:** 2022-01-06

**Authors:** Xian Zhang, Jiajia Ge, Xuejuan Zhu, Haifeng Zhang, Yuanzi Wang, Tongtong Xu, Wei Jiang, Bin Zhang

**Affiliations:** ^1^Nantong University, China; ^2^Affiliated Hospital of Nantong University, China

## Abstract

The aim of the present study was to investigate the effects and mechanism of oxymatrine (OMT) combined with compound yinchen granules (CYG) on the apoptosis of hepatocytes through the Akt/FoxO3a/Bim pathway in rats with acute liver failure. The rat model of acute liver failure was established using lipopolysaccharide/D-galactosamine (LPS/D-GalN). The expression of proteins in rat liver tissues was detected by western blot analysis. The mRNA expression of FoxO3a, Bim, Bax, Bcl-2, and caspase-3 in rat liver tissues was detected by RT-qPCR. The apoptosis rate of rat hepatocytes was determined by flow cytometry. Western blots showed that when compared with the normal group, the expression of p-Akt and p-FoxO3a in the model group was decreased (*P* < 0.05), while the expression of Bim was increased (*P* < 0.01). Compared with the model group, the expression of p-Akt and p-FoxO3a in the OMT group and the OMT combined with CYG groups was increased (*P* < 0.05 or *P* < 0.01), while the expression of Bim was decreased (*P* < 0.05). The Bax/Bcl-2 ratio and caspase-3 protein expression in the model group were significantly higher than those in the normal group (*P* < 0.01). The Bax/Bcl-2 ratio and the expression of caspase-3 protein in the OMT group and the OMT combined with CYG groups were significantly lower than those in the model group (*P* < 0.01). The results of RT-qPCR were consistent with those of western blot. The results of flow cytometry showed that the apoptosis rate of hepatocytes in the OMT group and the OMT combined with CYG groups was significantly lower than that in the model group (*P* < 0.05 or *P* < 0.01). We concluded that LPS/D-GalN can induce apoptosis of hepatocytes in rats with acute liver failure through the Akt/FoxO3a/Bim pathway. OMT combined with CYG inhibits apoptosis of hepatocytes in rats with acute liver failure via the Akt/FoxO3a/Bim pathway.

## 1. Introduction

Hepatocyte apoptosis plays an important role in the pathogenesis of acute liver failure (ALF) [1–3]. However, the mechanism of hepatocyte apoptosis in ALF is not yet fully understood. Moreover, there are no effective drugs to inhibit the apoptosis of hepatocytes. Therefore, it is important to identify effective therapeutic drugs and their targets. Due to the complicated etiology of hepatocyte apoptosis in ALF, it is unlikely to be treated by a single factor, and a multifactor comprehensive treatment is necessary. Oxymatrine (OMT) is an alkaloid extracted from the root of *Sophora flavescens*, *Sophora*, and *Radix alopecuroides*. Pharmacological and clinical trials have proved that OMT has anti-inflammatory, antiallergic, antifiber, antitumor, antiapoptosis, and other pharmacological effects [4–8]. Compound yinchen granules (CYG) are composed of yinchen (Herba artemisiae), danshen (Salvia miltiorrhiza), and dahuang (rhubarb). Yinchen has many functions such as protecting the liver and gallbladder and relieving jaundice and anti-inflammatory and analgesic effects and can reduce apoptosis and the expression of caspase-3 mRNA [9–11]. Danshen has anti-ischemic activity, dilates blood vessels, and reduces toxic liver injury and has antifibrosis and other pharmacological effects [12–15]. Dahuang has a variety of biological activities, such as antibacterial, anti-inflammatory, antioxidant, and liver and kidney protection, and can reduce the absorption of endotoxins in order to reduce liver cell damage in ALF [16–18]. In this study, lipopolysaccharide/D-galactosamine (LPS/D-GalN) was used to establish the ALF rat model. RT-qPCR, western blot, and flow cytometry were used to investigate the molecular effect and mechanism of OMT combined with CYG on the apoptosis of hepatocytes in rats with ALF in order to add new therapeutic targets for the treatment of hepatocyte apoptosis in ALF. This study will provide an experimental basis for OMT combined with CYG to inhibit liver cell apoptosis and identify effective drugs for the prevention and treatment of ALF.

## 2. Materials and Methods

### 2.1. Reagents and Antibodies

LPS and D-GalN were purchased from Sigma. CYG is composed of Herba artemisiae, Salvia miltiorrhiza, and rhubarb and was purchased from Jiangyin Tianjiang Pharmaceutical Co., Ltd. OMT was purchased from Shaanxi Baoji Fangsheng Biological Development Co., Ltd., with a purity of >98%. Akt, p-Akt^ser473^, FoxO3a, p-FoxO3a, Bim, Bax, Bcl-2, and active-caspase 3 antibodies were purchased from Abcam Corporation, USA. TRIzol was purchased from Shanghai Pufei Biotechnology Co., Ltd. PCR primers were designed and synthesized by Shanghai Ruiqiang Biotechnology Co., Ltd. The apoptosis detection kit was purchased from Biouniquer, USA.

### 2.2. Treatment of Animals

Sprague-Dawley (SD) rats, male, clean grade, aged 10-12 weeks, weighing 220 ± 20 g were provided by the Experimental Animal Center of Nantong University, license number: SYXK (su) 2012-0031. Ninety SD rats were randomly divided into the following six groups after one week of adaptive feeding: normal group, model group, OMT group, and OMT combined with CYG low, medium, and high dose groups, with 15 rats in each group. In CYG, the ratio of Herba artemisiae, Salvia miltiorrhiza, and rhubarb was 2 : 1 : 1. The ratio of low, medium, and high doses of CYG was 1 : 2 : 4, and the medium dose was equivalent to the clinical dose (6 g/kg/d). CYG was prepared with ddH_2_O and then administered to the animals. The groups and doses are shown in Table 1. Normal saline (NS) was injected intraperitoneally in the normal group and model group for three consecutive days. In the OMT group, OMT was injected intraperitoneally, once every day, at a dose of 2 ml/kg. The CYG low, medium, and high dose groups were given preconfigured CYG irrigation, and OMT at the same dose was injected 2 h later. In addition to the normal group, rats in the other groups fasted for 24 h and received a subcutaneous injection of LPS 10 *μ*g/kg and intraperitoneal injection of D-GalN 700 mg/kg to prepare the rat model of liver failure. Rats were sacrificed at 24 h after LPS/GalN injection. Liver samples were collected for further assessment. Disinfected scissors were then used to quickly cut the right outer lobe of the liver approximately 5 cm × 5 cm in size, and the liver tissue was placed in a frozen storage tube. The frozen tube was quickly placed in liquid nitrogen. When all rat liver tissues were removed, the frozen tubes in the liquid nitrogen tank were stored at -70°C.

### 2.3. RT-qPCR

Total RNA was extracted from liver tissue and quantified by an Eppendorf nucleic acid protein analyzer. RNA reverse transcription was performed according to the instructions, and RT-qPCR was performed. In the experiment, three wells were included for each sample, and three negative controls without samples were also included. All calculations were performed using the average cycle threshold value (Ct value) obtained from the three wells for each sample, and the difference between the *Q* values of the three wells was less than one cycle. Statistical analysis was also performed.

### 2.4. Western Blotting

One hundred mg liver tissue was weighed, cut into small pieces and washed in 1 ml precooled PBS (containing 0.1 mm PMSF), and repeated three times to remove red blood cells. One ml of precooled protein lysate (including 1 mM PMSF) was added to the ice bath homogenate until fully cracked. Protein concentration was detected by the BCA method, and western blot analysis was repeated three times. The gray analysis was performed by ImageJ software, and GraphPad Prism 5 software was used to analyze and count the expression levels of each group of characteristic proteins (with *β*-actin as the internal reference). Akt and FoxO3a showed the relative expression of the target protein by the ratio of the phosphorylated target protein to the total target protein, and then, SPSS 21.0 was used for statistical analysis.

### 2.5. Flow Cytometry

A 0.2 g sample of middle liver tissue was obtained. A single-cell suspension was prepared as follows: the liver tissue was cut into small pieces and placed in a grinder. NS was added, and the tissue was ground to a homogenate, and the grinder was repeatedly rinsed with NS to collect cells and filter out cell clusters. The cells were centrifuged for 5 min at 800 r/min, washed with PBS three times, and centrifuged for 5 min at 500 r/min, and the supernatant was discarded. The cells were resuspended in 250 *μ*l loading buffer, and 2.5 *μ*l Annexin V-FITC was added and gently mixed. The cells were incubated at room temperature and away from light for 10 min and centrifuged at 500 r/min for 5 min, and the supernatant was discarded. A total of 250 *μ*l of loading buffer was then added to resuspend the cells followed by the addition of 2.5 *μ*l of PI. The cells were placed in an ice bath away from light, and flow cytometry was performed within 1 hour.

### 2.6. Immunohistochemistry

A portion of middle liver tissue was cut into 6 mm × 5 mm × 5 mm pieces and immersed in 10% neutral formaldehyde solution for fixation for HE staining and immunohistochemical analysis. The tissue blocks were fixed for 24 h and prepared into paraffin sections according to the steps of dehydration, transparency, dipping, embedding, and sectioning. The protein expressions of active-caspase-3, Bax, and Bcl-2 in liver tissue were observed by SP staining. Grade according to the Formwitz method.

### 2.7. Statistical Analysis

The experimental data are expressed as the mean ± standard deviation ($\bar{x}\pm s$), and SPSS21.0 statistical software was used for data analysis. One-way analysis of variance was used to compare various means. The chi-squared test was used for comparisons between groups.

## 3. Results

### 3.1. General Observation and Liver Function

During the experiment, six rats died in the model group, one rat died in the OMT group, and no rats died in the OMT and CYG groups. In the model group, rat livers were obviously swollen and hyperemic with diffuse bleeding on the surface and large petechiae. The levels of ALT and AST in the normal group were 25 ± 12 IU/l and 35 ± 15 IU/l, respectively, while those in the LPS/D-GalN group were 2563 ± 587 IU/l and 2476 ± 525 IU/L, respectively. Compared with the normal group, it was significantly increased (*P* < 0.05, *P* < 0.05). ALT and AST levels in the OMT group were 1709 ± 344 IU/l and 1732 ± 345 IU/l, respectively, which were lower than those in the model group (*P* < 0.05, *P* < 0.05). The levels in the OMT+CYG-H+LPS/D-GalN group were 698 ± 252 IU/l and 639 ± 231 IU/l, respectively, which were further decreased compared with the OMT group (*P* < 0.05, *P* < 0.05).

### 3.2. The Expressions of p-Akt, Akt, p-FoxO3a, FoxO3a, Bax, Bcl-2, Caspase-3, and Bim Proteins in Rat Liver Tissues Were Detected by Western Blot Analysis

#### 3.2.1. Expression of Akt and p-Akt in Rat Liver Tissues in Each Group

**Figure 1:**
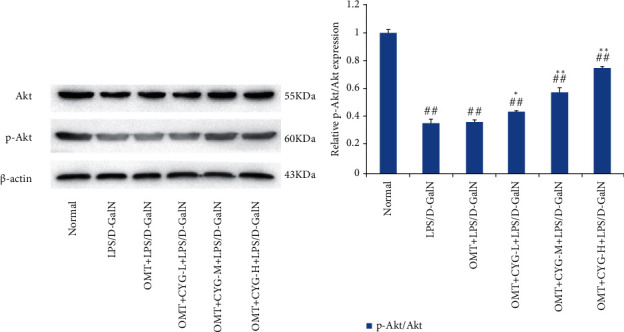
Expression of Akt and p-Akt protein in liver tissues of rats in each group. (a) Protein development diagram of Akt and p-Akt. (b) Statistical graph of transformed data. ^##^*P* < 0.01, compared with normal group; ^∗^*P* < 0.05,  ^∗∗^*P* < 0.01, compared with the model group.

The results of western blot analysis showed that Akt and p-Akt were expressed in the normal group. Compared with the normal group, the expression of p-Akt protein in the model group was significantly reduced (*P* < 0.01). Compared with the model group, the expression of p-Akt protein in all dose groups of OMT combined with CYG increased (*P* < 0.05 or *P* < 0.01), and the ratio of p-Akt/Akt increased with increasing CYG doses in the OMT+CYG groups within a certain dose range. The result is shown in Figure 1.

#### 3.2.2. Expression of FoxO3a and p-FoxO3a in Rat Liver Tissue

**Figure 2:**
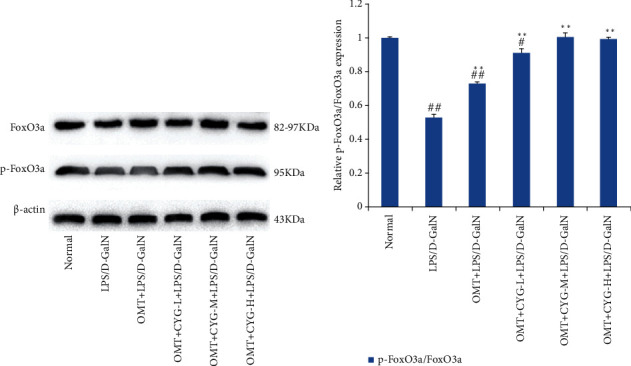
Expression of FoxO3a and p-FoxO3A in liver tissues of rats in each group. (a) Protein development diagram of FoxO3a and p-FoxO3a. (b) Statistical graph of transformed data. ^#^*P* < 0.05, ^##^*P* < 0.01, compared with the normal group; ^∗∗^*P* < 0.01, compared with the model group.

The results of western blot analysis showed that FoxO3a and p-FoxO3a proteins were expressed in the normal group. Compared with the normal group, the expression of p-Foxo3a protein in the model group was significantly reduced (*P* < 0.01). Compared with the model group, the expression of p-FoxO3a protein in the OMT and OMT+CYG groups was increased (*P* < 0.01). The result is shown in Figure 2.

#### 3.2.3. Bim Protein Expression in Rat Liver Tissue in Each Group

**Figure 3:**
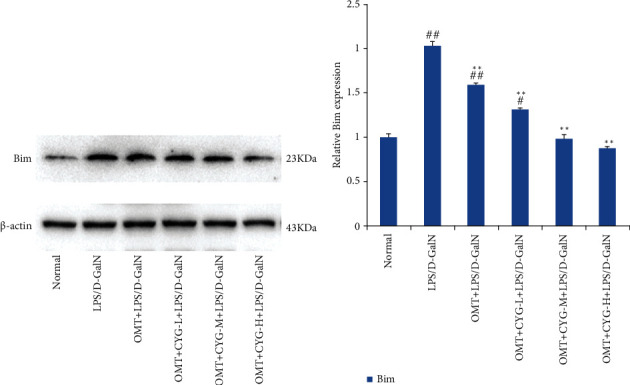
Bim protein expression in rat liver tissues of each group. (a) Bim protein development diagram of Bim; (b) statistical graph of transformed data. ^#^*P* < 0.05, ^##^*P* < 0.01, compared with normal group; ^∗∗^*P* < 0.01, compared with the model group.

Bim protein expression was observed in the liver tissues of rats in the normal group. Compared with the normal group, Bim protein expression in the model group was increased (*P* < 0.01). Compared with the model group, Bim protein expression in each dose group of the OMT and OMT+CYG groups was decreased (*P* < 0.01) and decreased with increasing CYG doses within a certain dose range. The result is shown in Figure 3.

#### 3.2.4. Expression of Bax, Bcl-2, and Caspase-3 Proteins in Rat Liver Tissues in Each Group

**Figure 4:**
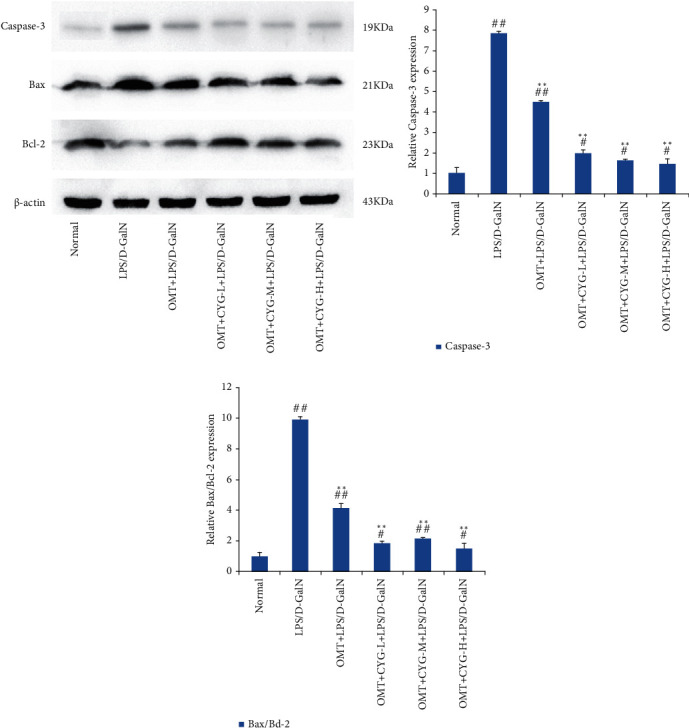
Expression of caspase-3, Bax, and Bcl-2 proteins in liver tissues of rats in each group. (a) Protein development diagram of caspase-3, Bax, and Bcl-2. (b, c) Statistical graph of transformed data. ^#^*P* < 0.05, ^##^*P* < 0.01, compared with normal group; ^∗∗^*P* < 0.01, compared with the model group.

The Bax/Bcl-2 ratio and caspase-3 protein expression were relatively low in the normal group and were significantly higher in the model group (*P* < 0.01). The Bax/Bcl-2 ratio and caspase-3 protein expression in the OMT and OMT+CYG groups were significantly lower than that in the model group (*P* < 0.01). The result is shown in Figure 4.

### 3.3. The Expressions of FoxO3a, Bim, Bax, Bcl-2, and Caspase-3 mRNA in Liver Tissues of Rats in Each Group

#### 3.3.1. The Expression of FoxO3a and Bim mRNA in Rat Liver Tissues in Each Group

**Figure 5:**
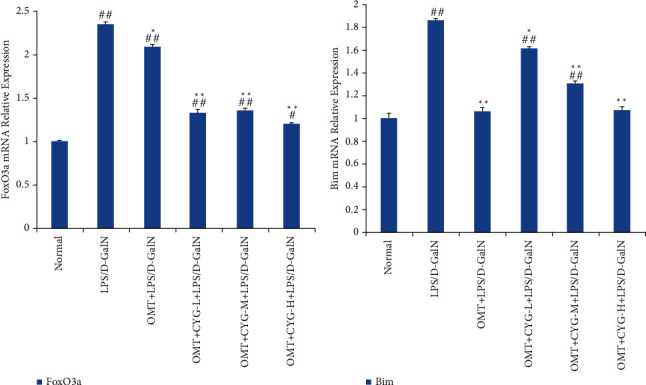
Expressions of FoxO3a and Bim mRNA in rat liver tissues of each group. ^#^*P* < 0.05, ^##^*P* < 0.01, compared with normal group; ^∗^*P* < 0.05,  ^∗∗^*P* < 0.01, compared with the model group.

The RT-qPCR results showed that the expression of FoxO3a and Bim mRNA in the model group was significantly increased compared with the normal group (*P* < 0.01). Compared with the model group, the expression of FoxO3a and Bim mRNA in the OMT and OMT+CYG groups was decreased (*P* < 0.05 or *P* < 0.01). The result is shown in Figure 5.

#### 3.3.2. The Expression of Bax, Bcl-2, and Caspase-3 mRNA in Rat Liver Tissues in Each Group

**Figure 6:**
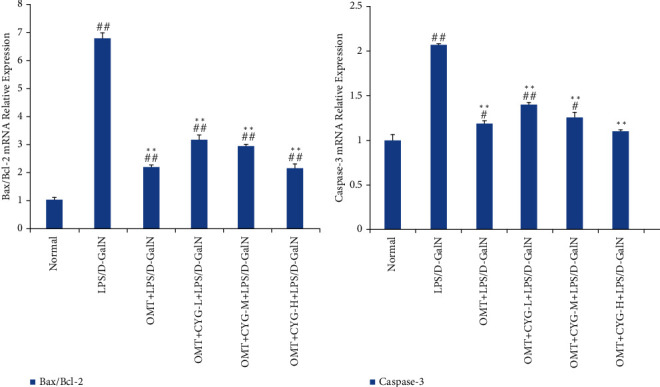
Expressions of Bax, Bcl-2, and caspase-3 mRNA in liver tissues of rats in each group. ^#^*P* < 0.05, ^##^*P* < 0.01, compared with normal group; ^∗∗^*P* < 0.01, compared with the model group.

Compared with the normal group, the Bax/Bcl-2 mRNA ratio in the model group was significantly increased (*P* < 0.01), and the Bax/Bcl-2 mRNA ratio in the OMT and OMT+CYG groups was significantly reduced (*P* < 0.01). The expression of caspase-3 mRNA in the model group was significantly higher than that in the normal group (*P* < 0.01). The expression of caspase-3 mRNA in all dose groups of OMT and OMT+CYG was significantly lower than that in the model group (*P* < 0.01). The result is shown in Figure 6.

### 3.4. Flow Cytometry Was Used to Detect the Rate of Hepatocyte Apoptosis in Each Group

**Figure 7:**
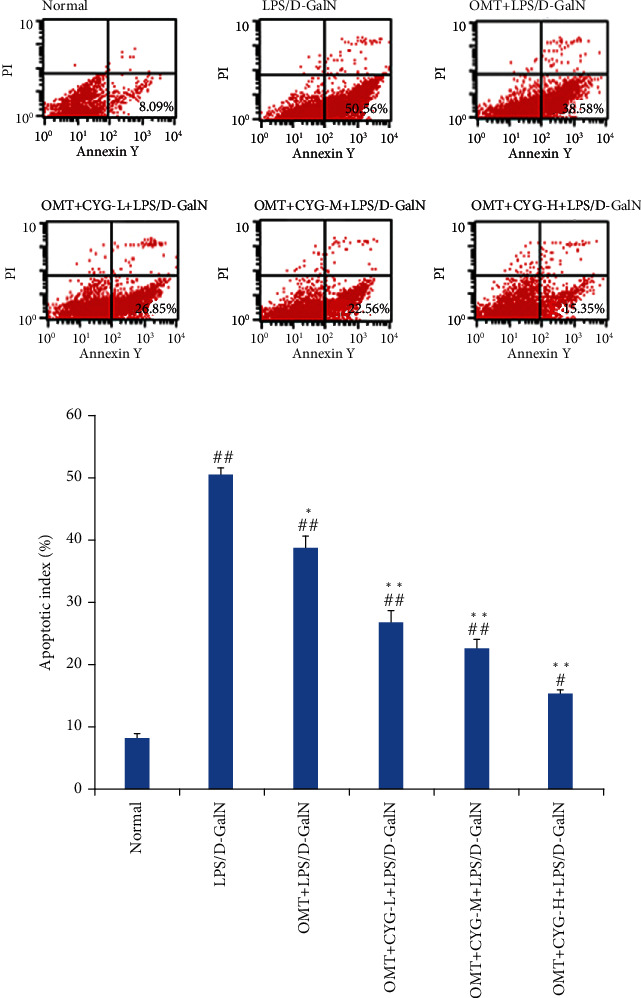
Apoptosis of rat hepatocytes in each group. (a) Flow cytometry quantitative analysis diagram; (b) statistical graph of transformed data. The lower left quadrant is normal living cells (LL), the upper left quadrant is necrotic cells (UL), the lower right quadrant is early apoptotic cells (LR), and the upper right quadrant is late apoptotic cells (UR). The total apoptotic cells were the sum of UR+LR. ^##^*P* < 0.01, compared with normal group; ^∗^*P* < 0.05,  ^∗∗^*P* < 0.01, compared with the model group.

The results of flow cytometry showed that the rate of hepatocyte apoptosis in the model group was significantly higher than that in the normal group (*P* < 0.01). Compared with the model group, the rate of hepatocyte apoptosis in the OMT and OMT+CYG groups was significantly reduced (*P* < 0.05 or *P* < 0.01). Within a certain dosage range, the effect of reducing the rate of apoptosis showed an increasing trend with increased CYG dosage. The result is shown in Figure 7.

### 3.5. Expression of Active-Caspase-3, Bax, and Bcl-2 in Liver Tissue

**Figure 8:**
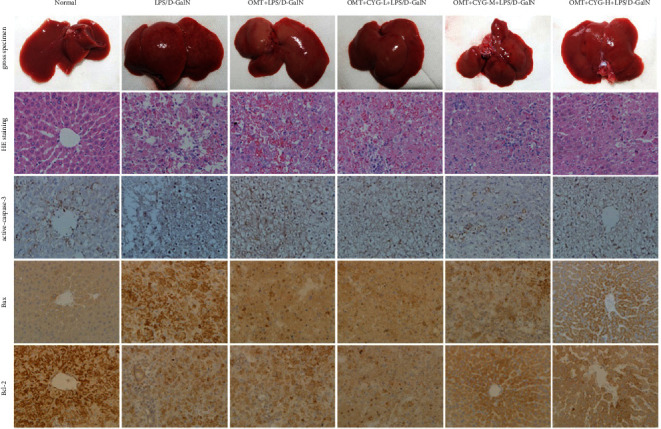
Expression of active-caspase-3, Bax, and Bcl-2 in liver tissue.

The expression of active-caspase-3 was relatively low in the normal group but significantly increased in the nucleus and cytoplasm of the model group. OMT could downregulate the expression of active-caspase-3, and CYG could further downregulate the expression of active-caspase-3. A small amount of Bax expression was found in the normal group, and the cytoplasm was pale yellow. The expression of Bax in the model group was significantly increased and brownish yellow. OMT could downregulate the expression of Bax, and CYG could further downregulate the expression of Bax. Bcl-2 showed obvious granules in the normal group and decreased granules in the model group. OMT could upregulate the expression of Bcl-2, and CYG could further upregulate the expression of Bcl-2, as shown in Figure 8.

## 4. Discussion

The main pathological changes in ALF are massive liver cell death and massive necrosis of liver tissue. It has been found that apoptosis is one of the main forms of hepatocyte death in ALF, and the occurrence of ALF is closely related to apoptosis of hepatocytes [19, 20]. In this study, flow cytometry was used to detect the rate of hepatocyte apoptosis in each treatment group. It was found that the apoptotic rate of rat hepatocytes in the model group induced by LPS/D-GalN was significantly higher than that in the normal group. The apoptotic rate in the OMT and OMT combined with CYG groups was significantly lower than that in the model group, and the decreasing effect of the apoptosis rate increased with increasing doses of CYG within a certain dose range. These results suggested that OMT combined with CYG could significantly reduce apoptosis of rat hepatocytes in ALF induced by LPS/D-GalN.

The mechanism of hepatocyte apoptosis is very complex and is regulated by many signal transduction pathways. In recent years, it was found that Toll-like receptor 4 (TLR4) expression is increased in liver tissue after liver injury and ALF and is involved in mediating multiple signaling pathways [21, 22]. The TLR4-mediated Akt/FoxO3a/Bim signaling pathway is an intracellular signal transduction pathway associated with cell proliferation, differentiation, and apoptosis [23]. Akt is a serine/threonine protein kinase B (protein kinase B, PKB). Akt activation can induce FoxO3a phosphorylation and transfer p-FoxO3a from the nucleus to the cytoplasm, and p-FoxO3a then cannot play the role of transcription factor (loss of apoptosis promoting activity). When the Akt activity declines, the FoxO3a phosphorylation level drops; nonphosphorylated FoxO3a is transferred to the nucleus with the help of the nuclear shuttle system, exerting its transcription factor effects, thus inducing the transcription of target genes and increasing the expression of proapoptotic protein Bim [24, 25]. As a member of the Bcl-2 proapoptotic family, Bim is an important apoptotic regulatory protein. As a transcription factor, FoxO3a can combine with the Bim promoter to upregulate the expression of Bim, which can promote the release of cytochrome c from mitochondria and activate caspase, leading to cell apoptosis [26, 27]. In this study, western blot analysis was used to detect p-Akt, Akt, p-FoxO3a, FoxO3a, and Bim proteins in rat liver tissue. It was found that Akt, p-Akt, FoxO3a, p-FoxO3a, and Bim proteins were expressed in normal rat liver tissue. The expression of p-Akt and p-FoxO3a in the model group was significantly lower than that in the normal group, while the expression of Bim protein in the model group was significantly higher than that in the normal group. The expression of p-Akt and p-FoxO3a proteins in the OMT and OMT combined with CYG groups at each dose was higher than that in the model group, while the expression of Bim protein was lower than that in the model group. The results of RT-qPCR showed that the expression of FoxO3a and Bim mRNA in the model group was significantly higher than that in the normal group. The expression of FoxO3a and Bim mRNA in the OMT group and OMT combined with CYG in each dose group was lower than that in the model group. These results suggested that p-Akt expression was decreased in rats in the model group, inactivated Akt could reduce the phosphorylation level of FoxO3a, and nonphosphorylated FoxO3a was translocated into the nucleus. FoxO3a, as a transcription factor, could induce the transcription of target genes [28], increase the expression of proapoptotic protein Bim, and thus increase the rate of rat hepatocyte apoptosis in the model group. The expression of p-Akt increased in the OMT and OMT combined with CYG groups at each dose, which increased the activity of Akt, and the expression of p-FoxO3a increased, which significantly reduced the expression of Bim, thus inhibiting the apoptosis of rat hepatocytes.

The Bcl-2 gene is closely related to apoptosis [29, 30]. The Bcl-2 gene family includes apoptotic suppressor genes and proapoptotic genes. Of these, Bax protein has an antiapoptotic effect against Bcl-2 protein. When the expression of Bax and Bcl-2 is balanced, cell survival is normal. When the expression of Bcl-2 is increased, the expression of Bax is not correspondingly increased, which could inhibit the apoptosis of cells. When the expression of Bax is increased, an isodimer is formed with Bcl-2, which could initiate the caspase enzyme cascade reaction to promote cell apoptosis. Therefore, the Bax/Bcl-2 ratio can be used as a reliable indicator for the detection of apoptosis. The caspases are a family of proteases closely related to apoptosis [31–33]. Caspase-3 is a key enzyme and executor of apoptosis [34, 35]. In this study, western blot analysis was used to detect Bax, Bcl-2, and caspase-3 proteins in rat liver tissue. It was found that the Bax/Bcl-2 ratio and caspase-3 protein expression were relatively low in the normal group. The Bax/Bcl-2 ratio and the expression of caspase-3 protein in the model group were significantly higher than those in the normal group. The Bax/Bcl-2 ratio and the expression of caspase-3 protein in all dose groups of OMT and OMT+CYG were significantly lower than those in the model group. The RT-qPCR results showed that the proportion of Bax/Bcl-2 mRNA in the model group was significantly higher than that in the normal group, while the proportion of Bax/Bcl-2 mRNA in the OMT and OMT+CYG groups was significantly lower than that in the model group. The expression of caspase-3 mRNA in the model group was significantly higher than that in the normal group, and the expression of caspase-3 mRNA in the OMT and OMT+CYG groups was significantly lower than that in the model group. These results showed that the Bax/Bcl-2 ratio and the expression of caspase-3 in the liver tissue of ALF rats were increased by LPS/D-GalN, thus increasing the apoptosis of hepatocytes. OMT and OMT+CYG can reduce the Bax/Bcl-2 ratio in ALF rat liver tissue, reduce the expression of caspase-3, and inhibit the apoptosis of liver cells.

Based on the above results, an Akt/FoxO3a/Bim signaling pathway is present in rat liver tissue. ALF induced by LPS/D-GalN can promote apoptosis of rat hepatocytes through the Akt/FoxO3a/Bim pathway. OMT combined with CYG can increase the expression of p-Akt and p-FoxO3a in ALF rat liver tissue and reduce the expression of proapoptotic proteins Bim and caspase-3 and the ratio of Bax/Bcl-2. OMT combined with CYG inhibits the apoptosis of ALF rat hepatocytes via the Akt/FoxO3a/Bim pathway.

## Figures and Tables

**Table 1:** Experimental animals in each group.

Group	Dosage (per kg)
Normal	NS
Model (LPS/D-GalN) OMT+LPS/D-GalN	10 *μ*g/700 mg 120 mg + 10 *μ*g/700 mg
OMT+CYG-L+LPS/D-GalN	120 mg + 3 g + 10 *μ*g/700 mg
OMT+CYG-M+LPS/D-GalN	120 mg + 6 g + 10 *μ*g/700 mg
OMT+CYG-H+LPS/D-GalN	120 mg + 12 g + 10 *μ*g/700 mg

## Data Availability

The datasets used and/or analyzed during the current study are available from the corresponding authors on reasonable request.
